# Exploring the nexus: Clinical and physiological correlation between cardiovascular disease and colorectal cancer

**DOI:** 10.1113/EP092898

**Published:** 2025-11-14

**Authors:** Mohamad Bashir, Ali Murtada, Matti Jubouri, Wael Awad, Ian Williams, Damian M. Bailey

**Affiliations:** ^1^ Neurovascular Research Laboratory, Faculty of Life Sciences and Education University of South Wales Pontypridd UK; ^2^ Department of Colorectal Surgery Liverpool University Hospital NHS Foundation Trust Liverpool UK; ^3^ Hull York Medical School University of York York UK; ^4^ Barts Heart Centre St Bartholomew's Hospital London UK; ^5^ Department of Vascular Surgery University Hospital of Wales Cardiff UK

**Keywords:** cardio‐oncology, cardiovascular mortality, colorectal cancer, comorbidity, molecular mechanism, physiology

## Abstract

Colorectal cancer (CRC) and cardiovascular disease (CVD) are leading causes of morbidity and mortality worldwide, traditionally studied as distinct pathologies. However, emerging evidence suggests a significant physiological and molecular overlap between these conditions, indicating that they might share common pathophysiological pathways. The aim of this paper is to explore the interconnected mechanisms linking CRC and CVD to identify shared risk factors, underlying molecular processes and potential avenues for integrated prevention and treatment strategies. The review highlights chronic inflammation, oxidative stress, metabolic dysregulation and gut microbiota dysbiosis as central factors contributing to CRC and CVD. Key inflammatory mediators, such as interleukin‐6, C‐reactive protein and tumour necrosis factor‐α, are discussed in the context of their dual role in tumour progression and atherogenesis. Additionally, metabolic disorders, including obesity, insulin resistance and hyperlipidaemia, are shown to elevate the risk of both diseases synergistically, with shared pathways involving insulin‐like growth factors and endothelial dysfunction. The manuscript also addresses the role of lifestyle and environmental factors, such as diet, physical activity and carcinogen exposure, in modulating the risk for CRC and CVD. Furthermore, it considers the implications of commonly used therapies, such as aspirin and statins, which exhibit cross‐benefits in both conditions. In conclusion, understanding the molecular and physiological crosstalk between CRC and CVD provides valuable insight into their co‐occurrence and offers opportunities for integrated screening, prevention and management approaches. This unified perspective supports the development of multidisciplinary strategies that could improve patient outcomes and reduce the global burden of these major chronic diseases.

## Abstract (highlights)

- **What is the topic of this review?**
  This review explores the molecular and physiological mechanisms linking colorectal cancer (CRC) to cardiovascular disease (CVD), emphasizing their shared pathophysiological pathways and risk factors. It underscores the clinical significance of this relationship for improving patient outcomes through integrated care approaches.
- **What advances does it highlight?**
  The review highlights advances in understanding how systemic inflammation, oxidative stress, cytokine signalling and endothelial dysfunction contribute to CRC progression and cardiovascular pathology. It also emphasizes the emerging role of colonic hypoperfusion, neutrophil extracellular traps and the gut microbiome in bridging CRC and CVD.

## 1 INTRODUCTION

An emerging concern is the direct correlation between colorectal cancer (CRC) and cardiovascular disease (CVD), particularly the mounting evidence that CRC patients have a higher burden of encountering an increased risk of cardiovascular mortality in comparison to the overall population. This lineage is a crucial consideration in the holistic management of CRC patients, because cardiovascular events can significantly impact the overall survival of this affected patient population (Strongman et al., 2019; Sturgeon et al., 2019).

The interrelationship between CRC and cardiovascular outcomes is multifaceted. It involves common and intertwined molecular and physiological pathways, risk factors, systemic effects of cancer, cardiotoxicity of cancer treatments and potential biological mechanisms linking these conditions. Understanding the connections between mechanistic and clinical intricacies is imperative for developing optimized medical and surgical management and treatment strategies, screening pathways, and follow‐up and community care that address oncological and cardiovascular health.

**FIGURE 1:**
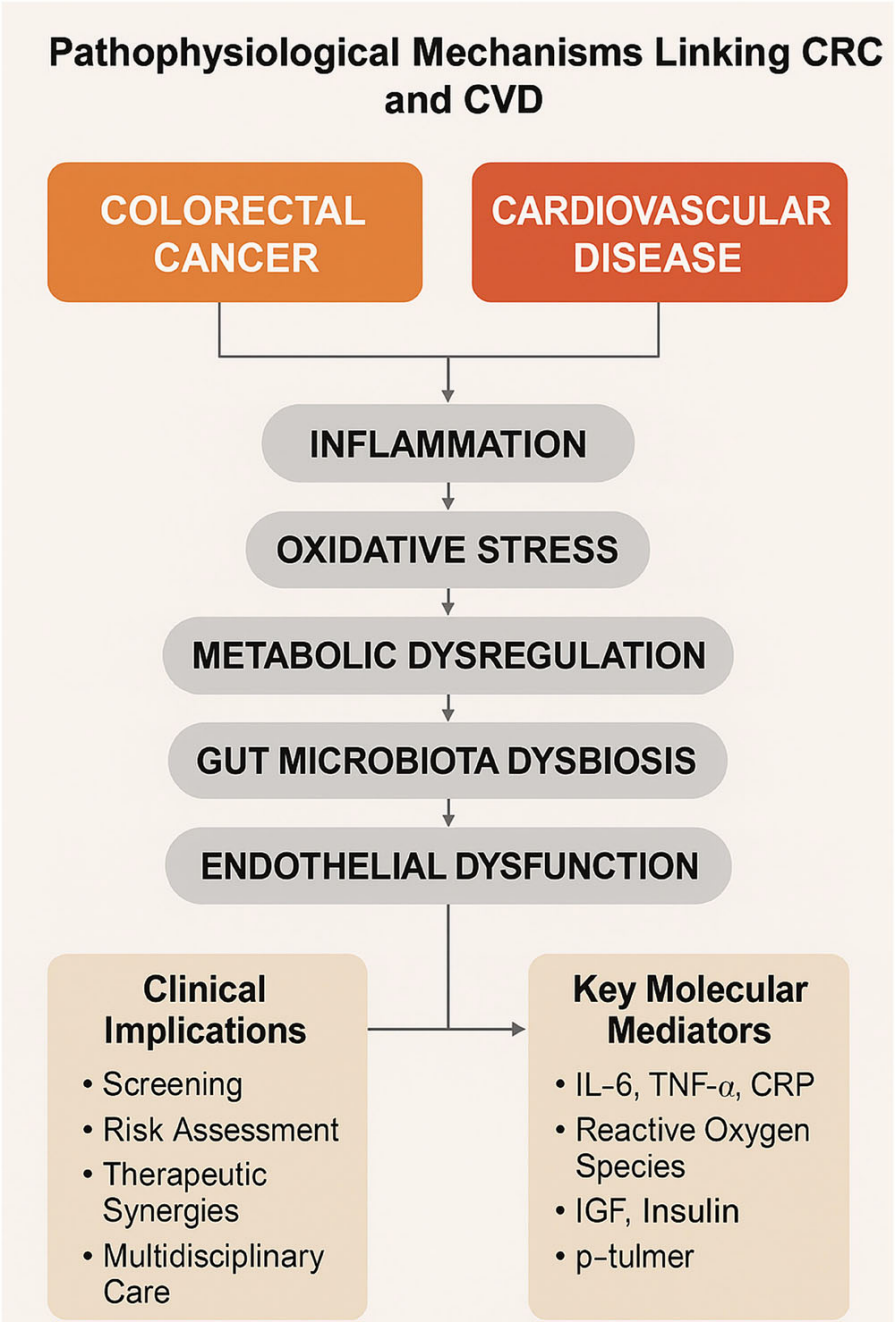
Schematic overview of the shared pathophysiological mechanisms linking CRC and CVD. Central overlapping pathways include inflammation, oxidative stress, metabolic dysregulation, microbiota dysbiosis and endothelial dysfunction. These mechanisms contribute to the development and progression of both diseases and have significant clinical implications for screening, risk assessment and integrated care. Abbreviations: CRC, colorectal cancer; CRP, C‐reactive protein; CVD, cardiovascular disease; IGF, insulin‐like growth factor; IL‐6, interleukin‐6; TNF‐α, tumour necrosis factor‐α.

The aims of this narrative review are to examine the evidence linking CRC to increased cardiovascular mortality comprehensively, to explore the potential underlying mechanisms (Figure 1), to evaluate the impact of cancer treatments on cardiovascular outcomes, to discuss clinical implications for patient management and to identify key areas for future research and directions.

## 2 EPIDEMIOLOGICAL EVIDENCE

**FIGURE 2:**
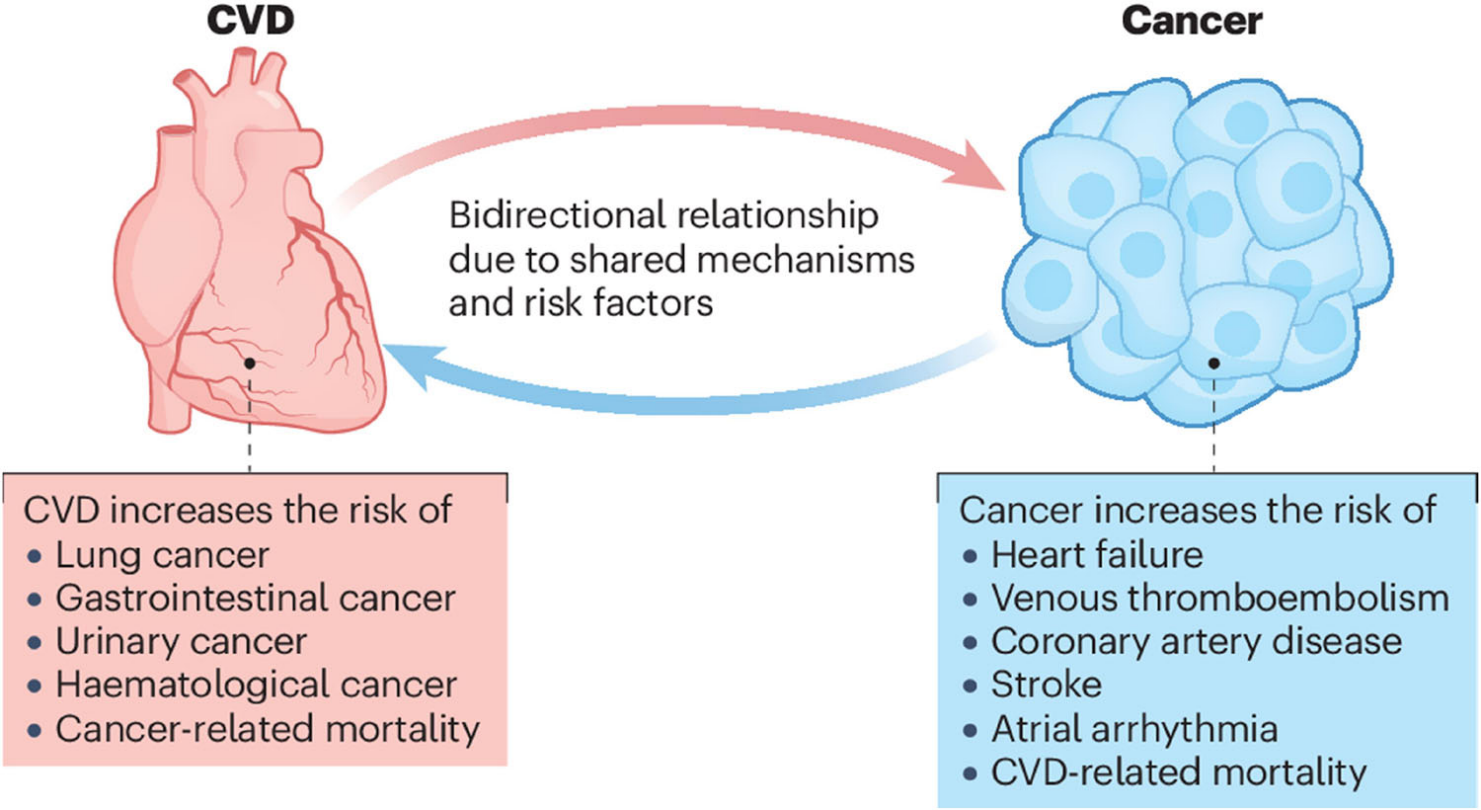
Illustration of the bidirectional relationship between CVD and cancer. Reused from Wilcox et al. (2024). Copyright permission obtained. Abbreviation: CVD, cardiovascular disease.

Epidemiological studies demonstrated a substantial prevalence of CVD in colorectal cancer patients in comparison to age‐matched control subjects without cancer. For instance, Strongman et al. (2019)), involving >100 000 cancer survivors with CRC, demonstrated a significantly elevated risk of cardiovascular untoward events, with hazard ratios ranging from 1.3 to 1.7 depending on the specific cardiovascular outcome examined. On a similar note, Sturgeon et al. (2019). found that CRC survivors had a 1.4‐fold increased risk of cardiovascular mortality compared with the general population, even after adjusting for traditional cardiovascular risk factors. The temporal relationship between CRC diagnosis and cardiovascular events is particularly noteworthy (Figure 2). Studies indicate that the risk is highest within the first year following a cancer diagnosis, with hazard ratios exceeding 2.0 for specific cardiovascular outcomes (Strongman et al., 2019; Sturgeon et al., 2019). However, elevated risk persists throughout the survivorship period, suggesting long‐term cardiovascular implications of CRC or its treatment (Zaorsky et al., 2017). A recent population‐based analysis of >630 000 CRC patients in the SEER database (2000–2021) confirmed this elevated cardiovascular risk, reporting a 16% higher CVD mortality compared with the general population. Importantly, this risk peaked within the first 2 years post‐diagnosis (≤45% increase) and was disproportionately higher amongst younger patients, Black patients and males (American College of Cardiology, 2025).

The clinical correlation between CRC and cardiovascular mortality reveals an important age‐related variation. It is reported that CRC patients <55 years old appear to have disproportionately higher relative risks of cardiovascular events compared with age‐matched control subjects (Schoormans et al., 2018). Although older patients have higher risks of cardiovascular morbidity owing to age‐related factors, the relative increase in risk of CRC is staggering in younger cohorts (Mehta et al., 2018). This pattern suggests that CRC might accelerate cardiovascular pathology, particularly in individuals who would otherwise have lower baseline cardiovascular risk.

Growing evidence indicates important sex‐specific differences in the relationship between CRC and cardiovascular outcomes. Several studies have observed that female CRC survivors experience higher relative increases in cardiovascular mortality compared with their male counterparts (Gernaat et al., 2017). These disparities might reflect differences in baseline cardiovascular risk profiles, hormonal factors, treatment approaches or biological susceptibilities to treatment‐related cardiotoxicity.

## 3 RISK PROFILE

**FIGURE 3:**
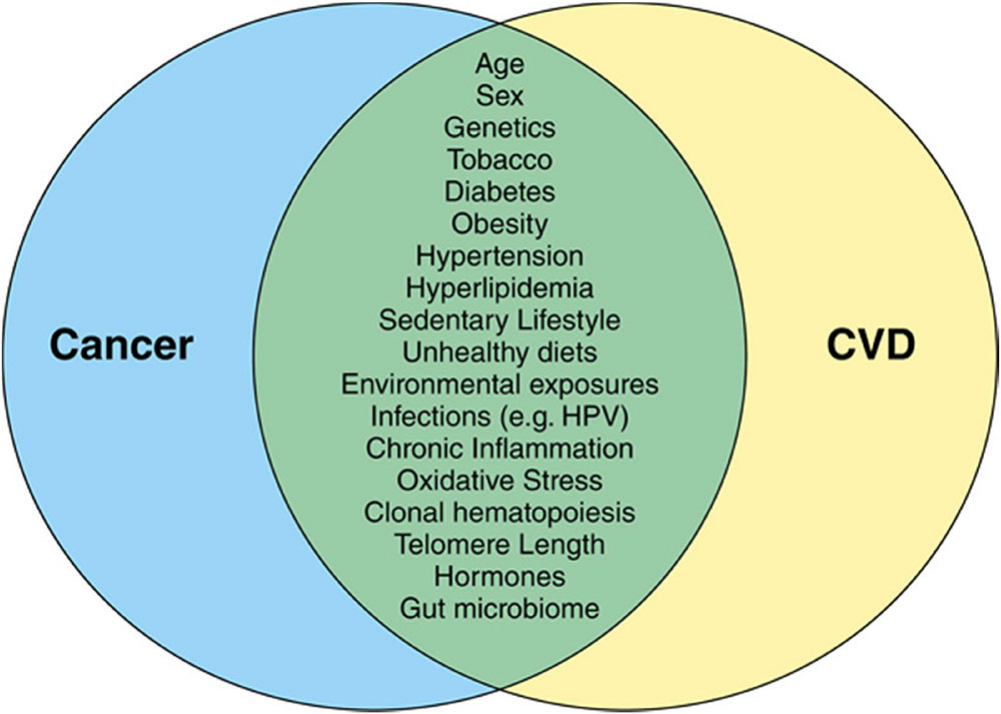
Venn diagram visualizing the shared risk factors between CVD and cancer. Reused from Handy et al. (2018). Published under open access. Copyright permission obtained. Abbreviations: CVD, cardiovascular disease; HPV, human papilloma virus.

The association between CRC and cardiovascular mortality is attributable, in part, to shared risk factors (Figure 3). Both conditions are strongly linked to lifestyle and metabolic factors, including obesity, physical inactivity, smoking, excessive alcohol consumption, poor dietary patterns and comorbid events. These include components of metabolic syndrome, such as hypertension, hyperlipidaemia and insulin resistance. The overlapping risk factors potentiate a high‐risk phenotype, predisposing patients to synchronous conditions and complicating efforts to establish the cause and effect between CRC and cardiovascular complications (Murphy et al., 2018).

Advanced‐stage disease (Stage III/IV) is associated with higher cardiovascular mortality than early‐stage disease, potentially reflecting more intensive treatment regimens, more significant systemic inflammation or increased metabolic derangements (Cespedes Feliciano et al., 2018).

It is worth noting that tumour location represents a common risk for CRC and is correlated with CVD. Emerging reports demonstrate that left‐sided colon cancers and rectal cancers might be associated with higher cardiovascular risk than right‐sided tumours. However, no underlying association has been made, and this matter remains at the centre of debate (Salem et al., 2017).

Moreover, the lack of a validated risk‐prediction model for CRC‐specific CVD represents an unmet clinical need. Clinically available risk‐prediction models misclassify significant proportions of cancer patients owing to the lack of integration of cancer‐specific variables, such as cancer stage, treatment protocols and biomarkers. Hence, the prediction performance is subtle and inaccurate.

Long‐term follow‐up studies of CRC survivors reveal important temporal patterns in cardiovascular risk. Although the immediate post‐diagnosis period (0–1 year) carries the highest relative risk, elevated cardiovascular mortality persists for at least 5–10 years after the initial cancer diagnosis (van Nimwegen et al., 2015). Armenian et al. (2017) demonstrated that the cumulative incidence of cardiovascular events continues to increase throughout survivorship, with no apparent plateau in risk even after 15 years of follow‐up.

## 4 COLORECTAL CARCINOMA: AN INITIATOR TO SYSTEMIC INFLAMMATION AND CYTOKINE NETWORKS

**FIGURE 4:**
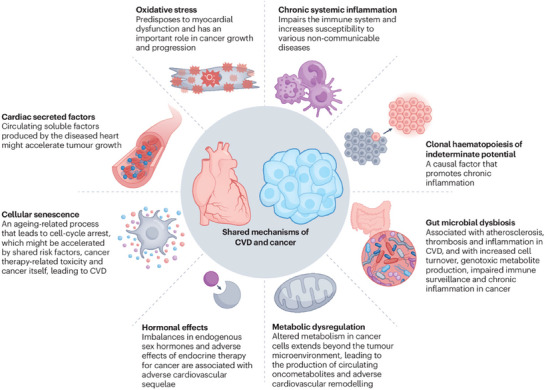
Illustration of shared mechanisms of CVD and cancer. Reused from Wilcox et al. (2024). Copyright permission obtained. Abbreviation: CVD, cardiovascular disease.

Colorectal carcinoma initiates a complex inflammatory cascade with inevitable cardiovascular implications and effects (Figure 4). Tumour cells and associated immune infiltrates produce elevated levels of pro‐inflammatory cytokines, including interleukin (IL)‐1β, IL‐6, IL‐8 and tumour necrosis factor‐α (TNF‐α) (Grivennikov et al., 2010). The pro‐inflammatory cascade amplifies inflammatory signalling via the activation of transcription factors, particularly nuclear factor kappa B (NF‐κB) and signal transducer and activator of transcription 3 (STAT3) (Hanahan & Weinberg, 2011). In response, endothelial dysfunction, vascular inflammation and accelerated atherosclerosis mechanisms are activated, disrupting cardiovascular integrity. Interleukin‐6, a key mediator in this process, induces C‐reactive protein (CRP) production by hepatocytes. Raised CRP levels are correlated with adverse cardiovascular outcomes in both general and cancer populations (Ridker et al., 2018). CRP directly impairs endothelial nitric oxide synthase function, reducing nitric oxide bioavailability and promoting endothelial dysfunction (Venugopal et al., 2002). Moreover, IL‐6 and TNF‐α upregulate endothelial adhesion molecules (VCAM‐1, ICAM‐1 and E‐selectin) that facilitate leucocyte recruitment to vascular walls, an early step in the development of atherosclerosis (Sprague & Khalil, 2009). CRC patients have increased circulating IL‐6 and TNF‐α compared with healthy individuals, promoting tumour burden on the cardiovascular system.

Inflammasomes, a multiprotein complex that activates caspase‐1 and promotes IL‐1β and IL‐18 secretion, have been explored, linking CRC to cardiovascular pathology. The NLRP3 inflammasome is activated by damage‐associated molecular patterns (DAMPs) released from injured colorectal epithelium and tumour cells (Karki & Kanneganti, 2019). Circulating DAMPs, including high‐mobility group box 1 and S100 proteins, are elevated in CRC patients and can trigger immune activation at distant vascular sites (Guzmán‐Ruiz et al., 2014). Single‐cell RNA sequencing has identified distinct populations of inflammasome‐activated monocytes in CRC patients that exhibit enhanced adhesion to endothelium and accelerated differentiation into foam cells when exposed to oxidized low‐density lipoprotein (Guo et al., 2015).

It suffices to deduce that the mechanism highlighted provides a direction between cancer‐induced inflammatory process and atherogenesis, leading to the strong correlation between CRC and CVD.

## 5 RESOLUTION OF INFLAMMATION AND DEFECTIVE REPAIR MECHANISMS

Not uncommonly, CRC patients have impaired inflammatory resolution and modulation pathways. The impairment can signal specialized pro‐resolving mediators derived from omega‐3 fatty acids, including resolvins, protectins and maresins, to orchestrate the resolution of inflammation and tissue repair (Serhan et al., 2014). Studies have reported reduced levels of these mediators in CRC patients, with concomitant decreases in their primary biosynthetic enzymes (15‐lipoxygenase and 12‐lipoxygenase) (Spite et al., 2014). The impairment in resolution pathways entices sustained vascular inflammation and impairs endothelial repair mechanisms. The evidence to support such an occurrence is derived from an experimental model that demonstrated that resolvin D1 and maresin 1 enhance endothelial barrier function, reduce leucocyte–endothelial interactions and promote clearance of apoptotic cells in atherosclerotic plaques (Fredman et al., 2016).

Therefore, it is important to understand that the relative deficiency of these mediators in CRC patients can potentially contribute to accelerated atherosclerosis and plaque instability. The culprit of the instability is clinically manifested in the setting of CVD.

## 6 OXIDATIVE STRESS AND REDOX IMBALANCE

CRC creates significant oxidative stress through multiple mechanisms, knowingly through altered metabolism, leading to increased aerobic glycolysis known as the Warburg effect. The process, in turn, leads to mitochondrial dysfunction and the release of reactive oxygen species (ROS) (Weinberg & Chandel, 2009). Infiltrating immune cells, particularly neutrophils and macrophages, generate substantial ROS through NADPH oxidase activation during respiratory bursts (Gao et al., 2007). As such, CRC patients have increased circulating oxidative stress markers, including malondialdehyde, F2‐isoprostanes and protein carbonyls, in comparison to healthy control subjects (Reuter et al., 2010). The antioxidant systems are compromised, with reduced levels of glutathione, superoxide dismutase, catalase and total antioxidant capacity observed in CRC patients (Valko et al., 2006). This pro‐oxidant state has profound cardiovascular implications. Increased ROS can lead to disintegration of the vascular endothelium through lipid peroxidation of membrane structures, oxidative modification of proteins and DNA damage (Montezano & Touyz, 2012).

It mediates the inactivation of nitric oxide, reduces vasodilatory capacity and promotes platelet aggregation and leucocyte adhesion (Förstermann et al., 2017). The process can upregulate pro‐inflammatory and prothrombotic genes, creating a self‐perpetuating cycle of vascular injury (Guzik & Touyz, 2017).

## 7 SPECIFIC OXIDATIVE PATHWAYS IN CANCER‐RELATED CARDIOVASCULAR INJURY

Multiple oxidative pathways have been implicated in cancer‐related cardiovascular injury. Myeloperoxidase, an enzyme expressed in neutrophils and macrophages, catalyses the formation of hypochloric acid and subsequent reactive species, leading to endothelial dysregulation and dysfunction. For instance, xanthine oxidase increases in cancer tissue and in the circulation of CRC patients, generating superoxide and hydrogen peroxide whilst depleting nitric oxide bioavailability (Battelli et al., 2016). NADPH oxidases or the so‐called NOX enzymes, particularly NOX1, NOX2 and NOX4 isoforms, are upregulated in the vascular endothelium of CRC patients (Pacher et al., 2006). Categorically, those enzymes produce superoxide as an end product and contribute directly to endothelial dysfunction, vascular inflammation and myocardial remodelling.

## 8 MITOCHONDRIAL DYSFUNCTION AND CELLULAR ENERGETICS

Mitochondrial dysfunction is an important area in which the research on cancer biology interacts with cardiovascular health. It is not an exception for CRC cells to have mitochondrial DNA mutations, anomalous respiratory chain complex activities and metabolic reprogramming, leading to ROS leakage from the electron transport chain (Pacher et al., 2006). One way that immune cells release cell‐free mitochondrial DNA into the circulation is as a viable source of a danger signal alarm (DAMP), which, in turn, activates Toll‐like receptor 9 and subsequently causes systemic inflammation (Wallace, 2012; Zhang et al., 2010). This statement is likely to indicate that other cancer‐related factors play a role in generating distant mitochondrial dysfunction via these mediators.

Detachment of several cancer cell lines from the primary tumour to colonize cardiac cells in distant tissues was displayed in experiments using photographs and live microscopy studies. The methods were focused on displaying the processes of cancer‐induced mitochondrial dysfunction in the context of myocardial tissue, including the role of specific cytokines (TNF‐α and IL‐1β), the supply of extracellular vesicles with regulatory microRNAs, and the metabolic reprogramming factors (hypoxia inducible factor‐1α and c‐Myc) (Tocchetti et al., 2019). These discoveries and mechanisms can be direct targets for preventing cancer‐related heart injury.

## 9 VASCULAR ENDOTHELIAL DYSFUNCTION AND REMODELLING

Endothelial dysfunction plays a crucial and pivotal role in atherogenesis, coupled with vascular remodelling, in the CRC patient population. CRC initiates endothelial activation that leads to increased expression of adhesion molecules, such as VCAM‐1, ICAM‐1 and E‐selectin. The process can also lead to reduced nitric oxide production and enhanced permeability. Takase et al. (1998) studied this process and concluded that flow‐mediated dilatation impairs endothelium‐dependent vasodilatation in CRC patients compared with matched population controls, with reductions proportional to tumour burden and inflammatory marker levels.

At the molecular level, vascular endothelial growth factor, produced by colorectal tumours, induces endothelial activation and increased permeability through vascular endothelial growth factor receptor 2‐mediated signalling cascades involving phospholipase C, protein kinase C and calcium mobilization (Ku et al., 1993). Other significant mediators of endothelial dysfunction in CRC include endothelin‐1, angiopoietin‐2 and various matrix metalloproteinases. On this note, Egan et al. (2008) demonstrated that endothelin‐1 levels are elevated two‐ to threefold in CRC patients compared with control subjects and are correlated with markers of endothelial activation. As such, Lerman et al. (1995) reflected that this potent vasoconstrictor impairs endothelium‐dependent vasodilatation, promotes leucocyte adhesion and stimulates smooth muscle cell proliferation, contributing to vascular remodelling, which can be detrimental and potentiates CVD. The way forward is to optimize a novel biomarker delineating intravascular injury. Mancuso et al. (2003) revealed that circulating endothelial cells detached from vessel walls owing to injury are elevated two‐ to fourfold in CRC patients compared with healthy control subjects. This biomarker has clinical implications, because the circulating endothelial cell counts are correlated with tumour volume, stage and subsequent cardiovascular events.

## 10 NEUTROPHIL EXTRACELLULAR TRAPS

Neutrophil extracellular traps (NETs) and NET‐mediated immunothrombosis progressively promote atherosclerosis and vessel plaque destabilization in CVD. They are considered causative of acute coronary syndrome. The resulting vascular compromise creates a hypoxic microenvironment that promotes genomic instability, chronic inflammation and dysbiosis within the colon. These conditions create a permissive environment for CRC initiation and progression. Furthermore, mounting evidence suggests that NETs might directly promote cancer cell proliferation, migration and invasion.

CRC patients demonstrate elevated plasma levels of NET markers, including cell‐free DNA, citrullinated histone H3 and myeloperoxidase–DNA complexes (Grilz et al., 2019). NET‐associated histones activate platelets and induce endothelial injury, whilst the DNA scaffold provides a surface for factor XII activation and intrinsic pathway initiation. Kapoor et al. (2018) reported that neutrophil elastase and other NET‐associated proteases also inactivate tissue factor pathway inhibitor and thrombomodulin, reducing anticoagulant capacity.

NETs significantly influence the tumour microenvironment and CRC cell behaviour. NET‐derived components, such as high mobility‐group box 1 and calprotectin, have been shown to enhance CRC cell proliferation through activation of Toll‐like receptor signalling and the receptor for advanced glycation end‐products, respectively. Furthermore, NET exposure promotes apoptosis resistance by upregulating anti‐apoptotic proteins, including Bcl‐2 and Bcl‐xL, thereby increasing tumour cell survival. NETs also enhance the migratory and invasive capabilities of CRC cells by establishing chemotactic gradients and releasing proteolytic enzymes that degrade extracellular matrices, facilitating tissue invasion.

In metastasis, NETs contribute to the entrapment of circulating tumour cells, shielding them from haemodynamic shear forces and immune clearance whilst promoting adhesion to distant endothelial sites, aiding metastatic seeding. Emerging data also indicate that NETs might underlie resistance to chemotherapy by activating prosurvival pathways within tumour cells and forming physical barriers that impede drug diffusion, highlighting their multifaceted role in CRC progression and therapeutic resistance.

## 11 THE COLONIC HYPOPERFUSION CULPRIT FOR COLORECTAL CANCER

A compelling hypothesis exists that correlates colonic hypoperfusion with colon cancer, enhanced and initiated by CVD. This relationship centres on colonic hypoperfusion, which results in reduced blood flow to the colonic tissues, resulting from cardiovascular pathology. The vascular supply of the colon includes branches of the superior and inferior mesenteric arteries, which can be compromised in patients with generalized atherosclerotic disease. Additionally, splanchnic circulation can be particularly vulnerable during states of reduced cardiac output or haemodynamic compromise that frequently accompany advanced coronary artery disease.

Understandably, the mechanism is confined to the pathophysiological process imposed by coronary artery disease. The potential effect of coronary artery disease leading to reduced cardiac output and redistribution of blood flow to a relatively low fraction of resistance areas occurs through the non‐diseased vasculature in the body. As such, compensatory redistribution of blood flows away from the splanchnic circulation to preserve cerebral and coronary perfusion. Additionally, coronary atherosclerosis often reflects a systemic process that simultaneously affects mesenteric vessels, directly compromising colonic blood supply and yielding a neurohormonal activation, which can inflict mesenteric vasoconstriction.

## 12 CLINICAL IMPLICATIONS: THE RIGHT COLON

An emerging pattern in the intersection between coronary artery disease and CRC is the stronger association observed with right‐sided (proximal) colon cancers compared with those in the left colon or rectum. Several anatomical and physiological factors might underlie this predilection. The right colon, especially the caecum, receives a relatively limited blood supply and experiences greater wall tension, making it more vulnerable to episodes of low perfusion. Additionally, this region operates in more anaerobic conditions, which could heighten its sensitivity to further reductions in oxygen availability.

Microbial composition also differs significantly across colonic regions; the right colon supports a distinct and more densely populated microbial community, including bacteria implicated in carcinogenesis, such as colibactin‐producing *Escherichia coli* and enterotoxigenic *Bacteroides fragilis*. Furthermore, right‐sided colorectal tumours exhibit unique molecular features, including a higher prevalence of microsatellite instability, the CpG island methylator phenotype and *BRAF* mutations. These characteristics might reflect chronic exposure to microbial toxins facilitated by barrier dysfunction in impaired perfusion. Together, these region‐specific factors contribute to a more nuanced understanding of the coronary artery disease–CRC connection and reinforce the plausibility of colonic hypoperfusion as a shared pathogenic mechanism.

## 13 DISCUSSION

Recent research has highlighted a compelling correlation between CVD and CRC, suggesting a multifaceted relationship driven by shared risk factors, overlapping pathophysiological mechanisms and possible direct interactions. Although historically considered distinct conditions, increasing epidemiological and mechanistic evidence supports that CVD and CRC might be interrelated components of broader systemic dysfunction.

**FIGURE 5:**
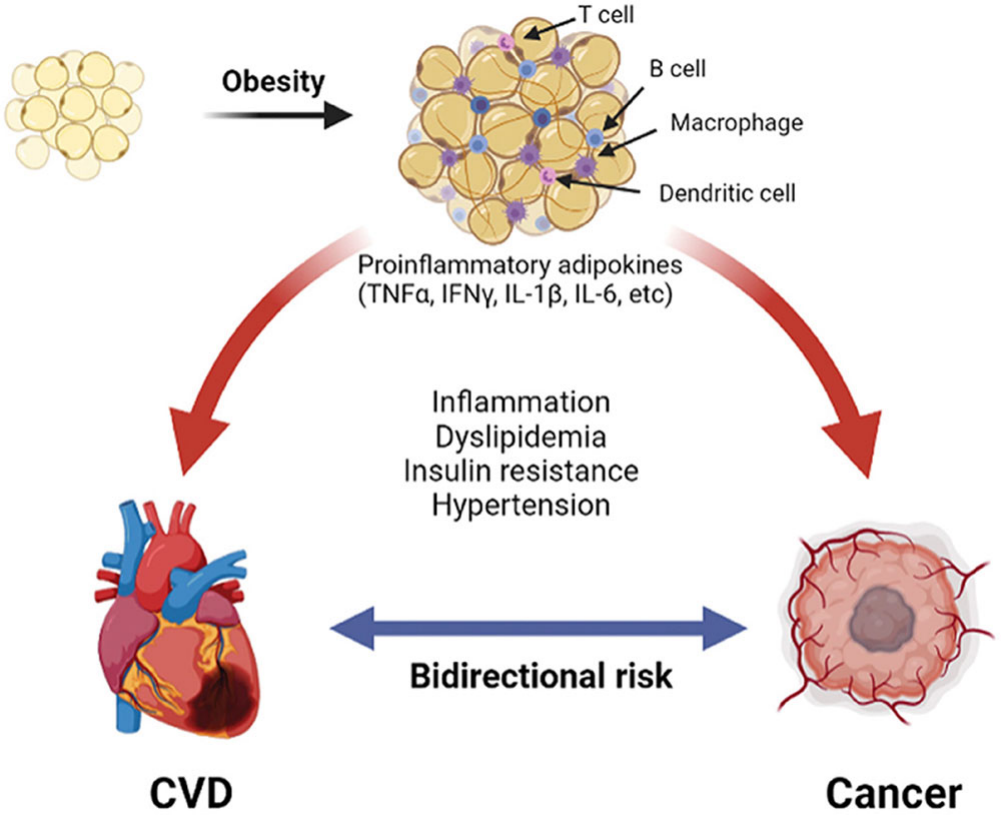
Metabolic syndrome and cancer. The pathological adipose tissue microenvironment has a reduction in vascularity with hypertrophy. This releases damage‐associated molecular patterns into the microenvironment, which trigger infiltration with the combination of pro‐inflammatory macrophages, dendritic cells, B and T cells. Additionally, there is an increase in pro‐inflammatory cytokines (e.g., TNFα, IFNγ and interleukins). Reused from Guha et al. (2021). Published under open access. Copyright permission obtained. Abbreviations: CVD, cardiovascular disease; IFNγ, interferon‐γ; IL‐1β, interleukin‐1β; IL‐6, interleukin‐6; TNF‐α, tumour necrosis factor‐α.

A key area of overlap lies in the prevalence of common modifiable risk factors. Obesity, type 2 diabetes, hypertension, dyslipidaemia, smoking, poor dietary patterns and sedentary behaviour are well‐established contributors to both atherosclerotic CVD and colorectal carcinogenesis. These factors often cluster within the context of metabolic syndrome, promoting chronic systemic inflammation, which is characterized by elevated levels of cytokines and acute‐phase reactants, such as IL‐6, TNF‐α and CRP. This inflammatory milieu has been implicated in vascular damage and tumour initiation, providing a biological basis for the observed association (Figure 5).

Beyond shared risk profiles, emerging data suggest direct mechanistic links between CVD and CRC. Colonic hypoperfusion, resulting from mesenteric atherosclerosis or cardiac insufficiency, can induce chronic ischaemia in the intestinal mucosa.

Repeated episodes of low‐grade ischaemia might impair epithelial barrier function, facilitating translocation of microbial products and promoting DNA damage, oxidative stress and genomic instability, all of which can contribute to neoplastic transformation. This mechanism might be particularly relevant to right‐sided (proximal) colon cancers, which are more vulnerable to ischaemia owing to their relatively tenuous vascular supply and higher baseline anaerobic environment. Complementary evidence from the UK Biobank and a meta‐analysis of 47 cohort studies (*n* ≈ 9.5 million) highlights the reverse relationship, showing that individuals with pre‐existing CVD carry a 13% higher overall cancer risk, with the strongest associations seen in those younger than 65 years (Shu et al., 2025). This bi‐directional interplay further reinforces the need for integrated surveillance strategies across oncology and cardiology.

The gut microbiome represents another potential axis of interaction. Dysbiosis, characterized by microbial diversity and function disruption, has been implicated in cardiovascular and colorectal disease. Specific microbial metabolites, such as trimethylamine *N*‐oxide, have been shown to contribute to atherosclerosis, and other microbial taxa have been associated with colorectal tumorigenesis via pro‐inflammatory or genotoxic activity. Thus, alterations in the gut microbial ecosystem might serve as a common underlying factor linking these conditions.

Additionally, pharmacological interventions for CVD might influence CRC risk. Owing to their lipid‐lowering and anti‐inflammatory properties, statins have demonstrated potential protective effects against CRC in observational studies. Likewise, low‐dose aspirin has been associated with reduced CRC incidence, probably through its anti‐inflammatory and anti‐platelet effects. Conversely, the impact of other cardiovascular medications, such as certain antihypertensives or antidiabetic agents, on CRC risk remains unclear and warrants further investigation.

Despite these associations, it is important to recognize the limitations of current evidence. Most findings are derived from observational studies, which are subject to confounding and cannot establish causality. Moreover, differences in screening practices, healthcare access and population characteristics might influence observed outcomes. Prospective, mechanistically oriented studies are essential to elucidate the nature and direction of this relationship.

In summary, the correlation between CVD and CRC appears to reflect a complex interplay of shared risk factors, systemic inflammation, impaired tissue perfusion and microbiome‐mediated pathways. Understanding these interconnections might inform integrated prevention, early detection and therapeutic intervention strategies across both disease domains.

## 14 CLINICAL IMPLICATIONS AND PRACTICE RECOMMENDATIONS

Understanding the intersection between CRC and CVD has crucial implications for patient care. Given that both conditions share overlapping risk factors and molecular pathways, a unified clinical approach offers opportunities for more effective prevention, screening and management.

Patients with CRC are at elevated long‐term risk of cardiovascular events, particularly within the first year post‐diagnosis and following exposure to cardiotoxic treatments, such as chemotherapy and radiation (Armenian et al., 2017; Wilcox et al., 2024). Cardiovascular risk assessment should therefore be integrated into the routine care of CRC patients, especially those with pre‐existing metabolic comorbidities or undergoing intensive therapy (Keramida et al., 2019).

Conversely, patients with established CVD or metabolic syndrome might benefit from earlier CRC screening, particularly if they are younger than the typical screening age or present with persistent gastrointestinal symptoms (Handy et al., 2018). The concept of ‘reverse screening’ (screening for cancer in CVD patients) has gained traction in recent prevention models (Wilcox et al., 2024).

Pharmacological agents such as statins and low‐dose aspirin are known to reduce cardiovascular risk and might confer protective effects against colorectal carcinogenesis via anti‐inflammatory, anti‐proliferative and anti‐platelet mechanisms (Drew & Chan, 2021; Elwood et al., 2021). Their dual utility supports broader implementation in eligible patients, particularly those with dual risk. Newer agents, such as SGLT2 inhibitors and GLP‐1 receptor agonists, which are being used increasingly in cardiovascular and metabolic medicine, are also being investigated for potential anti‐neoplastic properties (Wang et al., 2024).

Given the rising incidence of multimorbidity, particularly in ageing populations, multidisciplinary collaboration amongst oncologists, cardiologists, primary care physicians and nutritionists is essential (Keramida et al., 2019). The field of cardio‐oncology is expanding rapidly, but its principles should also be applied proactively in solid‐organ malignancies, such as CRC, not only in cardiotoxic chemotherapy settings. Integrated electronic medical records, co‐managed follow‐up plans and shared decision‐making models will be essential for managing risk holistically.

Current cardiovascular risk calculators (e.g., Atherosclerotic Cardiovascular Disease [ASCVD], Systematic Coronary Risk Evaluation 2 [SCORE2]) do not incorporate cancer‐specific variables, such as tumour stage, treatment history or systemic inflammation markers. There is a pressing need for oncology‐informed cardiovascular risk models tailored to CRC survivors (Jeong et al., 2022).

## 15 FUTURE DIRECTION

The link between coronary artery disease and CRC, especially when it comes to right‐sided tumours, is a fascinating area where cardiovascular and colorectal surgery intersect. It seems that colonic hypoperfusion could be a key player in connecting these two conditions, and there is growing evidence to back this up. When the blood flow to the colon is reduced, it can distort the epithelial barrier, ramp up inflammation, increase oxidative stress and alter the gut microbiome.

All these factors create a medium for the genetic and epigenetic changes that lead to CRC. The right side of the colon is particularly vulnerable to these issues, which helps to explain the patterns we see in epidemiological studies.

This emerging knowledge has real‐world implications for how we approach clinical care. It could lead to more tailored strategies for screening, preventing and assessing the risk of CRC. In addition, it emphasizes the need for a holistic approach to healthcare that recognizes the connections between different organ systems and disease processes.

## 16 CONCLUSION

In conclusion, this review underscores the intricate relationship between CRC and CVD, revealing shared risk factors and overlapping molecular and physiological mechanisms. Common factors, such as inflammation, oxidative stress and metabolic dysregulation, alongside lifestyle choices, contribute to the co‐occurrence of CRC and CVD. A deeper understanding of this interplay can pave the way for integrated strategies in screening, prevention and treatment, ultimately improving patient outcomes. Future research should focus on elucidating the precise mechanisms of colonic hypoperfusion in CRC development, particularly in right‐sided tumours. Clinically, this knowledge can drive more tailored screening and prevention strategies and a more integrated approach to patient care that acknowledges the interconnectedness of CRC and CVD.

## AUTHOR CONTRIBUTIONS

Mohamad Bashir: conceptualization, investigation, methodology, project administration, supervision, visualization, validation, writing–original draft preparation, writing–review & editing; Ali Murtada: investigation, methodology, writing–review & editing; Matti Jubouri: investigation, methodology, visualization, writing–original draft preparation, writing–review & editing; Wael Awad: project administration, writing–review & editing; Ian Williams: project administration, writing–review & editing; Damian M. Bailey: project administration, supervision, validation, writing–review & editing. All authors approved the final version of the manuscript and agree to be accountable for all aspects of the work in ensuring that questions related to the accuracy or integrity of any part of the work are appropriately investigated and resolved. All persons designated as authors qualify for authorship, and all those who qualify for authorship are listed.

## CONFLICT OF INTEREST

D.M.B. is Editor‐in‐Chief of *Experimental Physiology*, Chair of the Life Sciences Working Group, member of the Human Spaceflight and Exploration Science Advisory Committee to the European Space Agency and member of the Space Exploration Advisory Committee to the UK and Swedish National Space Agencies. D.M.B. is also affiliated to Bexorg, Inc. (USA), focused on the technological development of novel biomarkers of cerebral bioenergetic function and structural damage in humans.

## Data Availability

The evidence used to support this review is publicly available in electronic databases, such as PubMed, Ovid, Scopus and Embase.
